# Unlocking the Therapeutic Potential of a Manila Clam-Derived Antioxidant Peptide: Insights into Mechanisms of Action and Cytoprotective Effects against Oxidative Stress

**DOI:** 10.3390/foods13081160

**Published:** 2024-04-11

**Authors:** Hong Jin, Huishuang Zhao, Rui Shi, Fengjiao Fan, Wenjian Cheng

**Affiliations:** 1 College of Food Science, Fujian Agriculture and Forestry University, Fuzhou 350002, China; jinhong0921@163.com (H.J.); huishuangzhao1013@gmail.com (H.Z.); 2 College of Food Science and Technology, Huazhong Agricultural University, Wuhan 430070, China; 3 ChiBi Public Inspection and Testing Center, Xianning 437300, China; shirui923@163.com; 4 College of Food Science and Engineering, Nanjing University of Finance and Economics/Collaborative Innovation Center for Modern Grain Circulation and Safety, Nanjing 210023, China

**Keywords:** antioxidant peptide, Manila clam (*Ruditapes philippinarum*), oxidative stress, free radical scavenging, cytoprotection

## Abstract

Reactive oxygen species (ROS) are implicated in various pathological conditions due to their ability to induce oxidative damage to cellular components. In this study, we investigated the antioxidant properties of a peptide isolated from the hydrolysate of Manila clam (*Ruditapes philippinarum*) muscle. Purification steps yielded RPTE2-2-4, exhibiting potent scavenging activities against DPPH•, HO•, and O_2_•^−^, akin to Vitamin C. Structural analysis showed that the isolated peptide, LFKKNLLTL, exhibited characteristics associated with antioxidant activity, including a short peptide length and the presence of aromatic and hydrophobic amino acid residues. Moreover, our study demonstrated the cytoprotective effects of the peptide against H_2_O_2_-induced oxidative stress in HepG2 cells. Pretreatment with the peptide resulted in a dose-dependent reduction in intracellular ROS levels and elevation of glutathione (GSH) levels, indicating its ability to modulate cellular defense mechanisms against oxidative damage. Furthermore, the peptide stimulated the expression of the cytoprotective enzyme heme oxygenase-1 (HO-1), further reinforcing its antioxidant properties. Overall, our findings highlight the potential of the Manila clam-derived peptide as a natural antioxidant agent with therapeutic implications for oxidative stress-related diseases. Further investigation into its mechanisms of action and in vivo efficacy is warranted to validate its therapeutic potential.

## 1. Introduction

Reactive oxygen species (ROS), such as hydrogen peroxide, superoxide, and hydroxyl radicals, are natural by-products of metabolic processes within living organisms [1,2]. Oxidative stress arises when there is an imbalance between the production of ROS and the cell’s ability to neutralize them [3]. This imbalance can lead to damage to cellular macromolecules and is associated with numerous age-related chronic diseases, including cancer, Alzheimer’s disease, inflammation, and atherosclerosis [4,5,6]. In response to oxidative stress, the body employs endogenous antioxidant systems, including enzymes like superoxide dismutase, catalase, and glutathione peroxidase, as well as non-enzymatic compounds like selenium, α-tocopherol, and Vitamin C, to mitigate oxidative damage. However, the efficacy of these endogenous antioxidants may decline with age or due to different factors such as pollutants, fatigue, alcohol consumption, and dietary habits [7,8].

To bolster the body’s antioxidant defenses, there is growing interest in identifying new, safe, and effective antioxidants from food sources. In addition to well-known antioxidants like phenolic compounds and carotenoids, peptides with antioxidative properties have garnered attention in recent research [9,10,11]. Numerous studies have focused on isolating and characterizing peptides derived from various food proteins, including fish, shellfish, eggs, meat, and plant sources [12,13,14]. These peptides, typically consisting of 3–40 amino acid residues, have shown promising antioxidative potential in vitro and are being explored for their possible applications in functional foods and nutraceuticals [15,16,17,18]. Assaad Sila reviewed the isolation, identification, and application of antioxidant peptides from marine by-products in food systems [19]. Xiaoqian Zhang prepared antioxidant peptides from *Gracilariopsis lemaneiformis* proteins by hydrolyzing using different proteases (trypsin, pepsin, papain, α-chymotrypsin, alcalase) [20]. Furthermore, N. S. Sampath Kumar purified and identified antioxidant peptides from the skin protein hydrolysate of two marine fishes, horse mackerel (*Magalaspis cordyla*) and croaker (*Otolithes ruber*) [21]. These peptides have demonstrated potential in combating oxidative stress-related diseases and age-related conditions, such as cardiovascular diseases, cancer, and neurodegenerative disorders. Additionally, their natural origin from marine sources offers advantages such as sustainability, biodiversity, and bioactivity.

The Manila clam (*Ruditapes philippinarum*) is a commercially important species of marine shellfish in East Asia and the Mediterranean, prized for its nutritional value and taste [22]. While previous studies have highlighted various biological activities of peptides derived from Manila clam hydrolysates, such as anticancer, antibacterial, and anti-inflammatory effects [23,24,25], limited research has focused on evaluating their antioxidant properties. Therefore, this study aims to characterize the antioxidant peptide isolated from enzymatic hydrolysates of the Manila clam and investigate its antioxidative properties.

## 2. Materials and Methods

### 2.1. Materials

Manila clams were procured from a local market in Fuzhou, China. Trypsin with a nominal activity of 6 × 10^4^ U/g was sourced from Solarbio Co., Ltd. (Beijing, China). Chemicals and reagents, including 1,1-diphenyl-2-picrylhydrazyl (DPPH), 1,2,3-trihydroxybenzene, 1,10-phenanthroline, 2′,7′-Dichlorodihydrofluorescein diacetate (DCFH-DA), 5-Chloromethylfluorescein Diacetate (CMFDA), and 3-(4,5-dimethyl-thiazol-2-yl) -2,5-diphenyltetrazoliumbromide (MTT), were procured from Sigma (St. Louis, MO, USA). Dulbecco Modified Eagle Medium (DMEM), fetal bovine serum (FBS), penicillin-streptomycin, and 0.5% trypsin-EDTA were obtained from Gibco BRL (Fredrick, MD, USA). All chemicals and reagents used in this study were of analytical grade.

### 2.2. Preparation of Manila Clam Hydrolysate Using Trypsin

The fresh Manila clams were first shelled, and the viscera were removed. The clam muscle obtained was then rinsed with distilled water and homogenized by pounding. The homogenate was diluted with 10 mM phosphate buffer in a mass ratio of 1:5. Enzymatic hydrolysis was performed using trypsin with a substrate/enzyme ratio of 100:1 for 3.5 h at 50 °C and pH 7.5 [26]. The enzymatic reaction was terminated by heating the mixture for 10 min in a water bath at 95 °C. Subsequently, the hydrolysate was centrifuged at 8000 rpm for 15 min. The supernatant, referred to as RPTE, was then lyophilized using an LGJ-12S freeze dryer (Songyuan Vacuum Engineering, Huaxing Technology Development Co., Beijing, China) and stored at −20 °C for further analysis.

### 2.3. Isolation of Antioxidant Peptides from RPTE Hydrolysate

#### 2.3.1. Sephadex Gel Filtration Fractionation

The freeze-dried RPTE was dissolved in distilled water to achieve a concentration of 50 mg/mL, and then filtered through a 0.45 μm membrane. Subsequently, the solution was applied onto a Sephadex G-25 gel filtration column (1.6 × 50 cm) (Ruji Technology Development Co., Ltd., Shanghai, China), which had been pre-equilibrated with distilled water [27]. Elution was carried out using distilled water at a flow rate of 2 mL/min, while monitoring the eluate at 280 nm with a UV-spectrophotometer (Huxi Analysis Instrument Factory Co., Shanghai, China). Fractions were collected and subsequently lyophilized.

The free radical scavenging activities of these fractions against DPPH•, hydroxyl radical (HO•), and superoxide anion radical (O_2_•^−^) were assessed individually [28]. The fraction exhibiting the most potent antioxidative activities was subjected to further purification using a Sephadex G-15 gel filtration column (2.6 × 60 cm, Ruji Technology Development Co., Shanghai, China). Elution was performed with distilled water at a flow rate of 1.25 mL/min. The fractions (RPTE2-1–RPTE2-4) were combined and lyophilized. Based on the same free radical scavenging assays, the fraction demonstrating the strongest antioxidant activity was selected for additional purification steps.

#### 2.3.2. Reversed-Phase High Pressure Liquid Chromatography Fractionation

RPTE2-1 was dissolved in deionized and distilled water and filtered using a 0.45 μm membrane. Subsequently, it underwent purification via reversed-phase high-pressure liquid chromatography (RP-HPLC) on a Kromasil C18 column (4.6 × 150 mm, 5 μm, Akzo Nobel N.V, Amsterdam, The Netherlands) employing a linear gradient of acetonitrile (5–70% over 25 min) containing 0.1% trifluoroacetic acid (TFA) at a flow rate of 1.0 mL/min [29]. The eluate was monitored at 214 nm, and the separated fractions were collected and subsequently lyophilized. The efficacy of each fraction in scavenging free radicals, including DPPH, hydroxyl radical, and superoxide anion radical, was assessed to identify the most potent fraction.

### 2.4. Antioxidant Peptide Identification

To identify the antioxidative peptide, the antioxidant fraction obtained from the screening process was re-suspended in Nano-RPLC buffer A (0.1% FA, 2% ACN) [30]. Online Nano-RPLC was conducted using the Eksigent nano LC-UltraTM 2D System (AB SCIEX, Foster City, CA, USA). The sample was loaded onto a C18 nano LC trap column (100 μm × 3 cm, C18, 3 μm, 150 Å) and washed with Nano-RPLC buffer A at a flow rate of 2 μL/min for 10 min. The peptides were eluted using a linear gradient of 5–35% acetonitrile (0.1% formic acid) over 90 min. The gradient was applied to an analytical Chrom XP C18 column (75 μm × 15 cm, C18, 3 μm, 120 Å) with a spray tip.

Data acquisition was performed using a Triple TOF 5600 System (AB SCIEX, Foster City, CA, USA) equipped with a Nano spray III source (AB SCIEX, Foster City, CA, USA) and a pulled quartz tip as the emitter (New Objectives, Littleton, MA, USA). Ionization was achieved with an ion spray voltage of 2.5 kV, a curtain gas of 30 PSI, a nebulizer gas of 5 PSI, and an interface heater temperature of 150 °C. For information-dependent acquisition, survey scans were acquired in 250 ms, and up to 35 product ion scans were collected if these exceeded a threshold of 150 counts per s (counts/s) with charge states ranging from 2+ to 5+. The total cycle time was set to 2.5 s. A rolling collision energy setting was applied to all precursor ions for collision-induced dissociation (CID). Dynamic exclusion was set for 1/2 of the peak width (18 s), and the precursor ions were refreshed off the exclusion list.

Based on the MS and MS/MS spectra obtained, peptide identification was performed using the MASCOT V2.3 search engine (Matrix Science Co., Ltd., London, UK). The search parameters included the Ruditapes philippinarum database, trypsin as the digestion enzyme, ±15 ppm for precursor ion tolerance, and ±0.15 Da for-fragment ion tolerance.

### 2.5. Assessment of Free Radical Scavenging Activities

#### 2.5.1. DPPH Radical Scavenging Activity

The DPPH scavenging activity was evaluated following a method described by Pan [31], with slight modifications. Solutions of 2 mL containing varying concentrations of samples were mixed with 2 mL of a 0.2 mM ethanolic solution of DPPH•. Additionally, DPPH solution without the sample and sample solution without DPPH were prepared as controls. All prepared samples were then incubated in darkness for 30 min at room temperature. Subsequently, the absorbance of the samples, control, and blank was measured at 517 nm using a UV-Visible Spectrophotometer (Persee General Instrument Co., Ltd., Beijing, China). The DPPH scavenging activity was calculated using the following equation:

$$DPPH\;scavenging\;activity\left(\%\right)=\left(1-\frac{A_{i}-A_{j}}{A_{0}}\right)\times 100$$

where *A_i_*, *A_j_* and *A*_0_ represent the absorbance of the samples, blank, and control, respectively.

#### 2.5.2. Hydroxyl Radical (HO•) Scavenging Activity

The capacity to scavenge hydroxyl radicals was assessed following the methods outlined by Wang et al. [32]. Initially, a mixture was prepared by combining 1.5 mL of 5 mM 1,10-phenanthroline solution with 2 mL of phosphate buffer (pH 7.4). Subsequently, 0.5 mL of EDTA (15 mM) and 2 mL of the sample were added to the mixture separately, followed by the addition of 1 mL of FeSO_4_ (5 mM). The reaction was initiated by adding 1 mL of H_2_O_2_ (0.1%, *v*/*v*). Immediately after the addition of H_2_O_2_, the mixture was diluted with deionized and distilled water to a final volume of 10 mL, and then incubated at 37 °C for 1 h. The absorbance at 536 nm was measured. The hydroxyl radical scavenging activity was calculated using the following equation:

$$HO^{.}\;scavenging\;activity\left(\%\right)=\left(1-\frac{A_{i}-A_{m}}{A_{n}}\right)\times 100$$

where *A_i_* is the absorbance of the samples, *A_m_* is the absorbance of the non-damaged group without H_2_O_2_, and *A_n_* is the absorbance of the damaged group without the sample solution.

#### 2.5.3. Superoxide Anion (O_2_•^−^) Scavenging Activity

The O_2_•^−^ scavenging activity was determined following a method described by Yang et al. [33], with slight modifications. The reaction mixture comprised 2.5 mL of 50 mM Tris-HCl buffer (pH 8.2) and 2 mL of the sample at various concentrations. The mixture solutions were pre-incubated at 25 °C for 20 min. Subsequently, the reaction was initiated by adding 0.15 mL of 3 mM 1,2,3-trihydroxybenzene solution (dissolved in 10 mM HCl). The absorbance at 325 nm was recorded every 30 s for 4 min. The scavenging capacity of the O_2_•^−^ was calculated using the following equation:

$$O_{2}^{.-}scavenging\;activity\left(\%\right)=\left(1-\frac{A_{i}-A_{m}}{A_{n}}\right)\times 100$$

where *A_i_* is the absorbance without the sample, *A_m_* is the absorbance with the sample, and *A_n_* is the absorbance without the sample multiplied by a factor.

### 2.6. Cellular Antioxidative Activities

#### 2.6.1. Cell Culture

The human hepatocellular carcinoma (HepG2) cells were procured from the American Type Culture Collection (ATCC, Virginia, USA). These cells were cultured in Dulbecco Modified Eagle Medium (DMEM) supplemented with 10% fetal bovine serum (FBS) and a 1% antibiotic mixture of penicillin (100 U/mL) and streptomycin (100 μg/mL). Cultures were maintained under a humidified atmosphere at 37 °C with 5% CO_2_ in an incubator (Thermo Forma, Waltham, MA, USA), and incubated as followed time in different experiments.

#### 2.6.2. Cell Viability Measurement

Cell viability was assessed using the MTT assay, as previously described by Cho et al. [34], with slight modification. Briefly, after treatment, the culture medium was replaced with 100 μL of fresh medium containing 0.5 mg/mL of MTT and incubated at 37 °C for 1 h. Subsequently, the medium containing MTT was removed, and the reduced formazan dye was solubilized by adding 100 μL of dimethyl sulfoxide (DMSO) to each well. After gentle mixing, the absorbance was measured at 570 nm using an ELISA reader (Benchmark Plus; Bio-Rad, Hercules, CA, USA). Cell viability was expressed as the relative formazan formation in treated samples compared to control cells after correction for background absorbance.

#### 2.6.3. Intracellular Reactive Oxygen Species (ROS) Measurement

2′,7′-Dichlorodihydrofluorescein diacetate (DCFH-DA) is capable of entering cells and producing a fluorescent signal upon intracellular oxidation by reactive oxygen species (ROS), such as hydrogen peroxide and lipid peroxides. The resulting fluorescence can directly reflect the overall intracellular ROS concentration [35]. Briefly, the HepG2 cells were seeded in a 24-well plate at a density of 2 × 10^5^ cells/mL and incubated for 12 h. After treatment, the cells were incubated with 20 μM DCFH-DA for 30 min, followed by washing with serum-free medium three times to remove extracellular DCFH-DA. Subsequently, the fluorescence intensity was measured using a flow cytometer (Becton Dickinson, Franklin Lakes, NJ, USA). The fluorescence spectroscopy excitation/emission were set at 485 nm/535 nm.

#### 2.6.4. Measurement of Glutathione (GSH) Levels

Glutathione (γ-glutamyl-cysteinyl-glycine, GSH) is a non-protein thiol that plays a crucial role in intracellular defense against ROS-induced oxidative damage. The concentration of GSH was assessed using 5-Chloromethylfluorescein Diacetate (CMFDA). The HepG2 cells were seeded in a 24-well plate at a density of 2 × 10^5^ cells/mL and incubated for 12 h. Subsequently, the cells in different wells were treated with the peptide at concentrations of 5, 10, 25, 50, and 100 μM for 2 h, respectively. After treatment, H_2_O_2_ (750 μM) was added to the plates and incubated for an additional 24 h. The cells were then incubated with 50 μM CMFDA for 30 min and immediately measured using a flow cytometer (Becton Dickinson).

#### 2.6.5. Western Blot Analysis of Protein Expression

After harvesting and washing with PBS, the HepG2 cells were treated with lysis buffer containing 10% glycerol, 1% Triton X-100, 1 mM Na_3_VO_4_, 1 mM EDTA, 10 mM NaF, 1 mM Na_4_P_2_O_7_, 20 mM Tris buffer (pH 7.9), 100 μM β-glycerophosphate, 137 mM NaCl, and 5 mM EDTA, along with one Protease Inhibitor Cocktail Tablet (Roche, Indianapolis, IN, USA), on ice for 1 h. The lysates were then centrifuged at 10,000× *g* at 4 °C for 30 min. Protein concentrations were determined using the Bio-Rad protein assay (Bio-Rad Laboratories, Inc., Hercules, CA, USA).

Subsequently, samples containing 50 μg of protein were mixed with 5× SDS protein loading buffer, consisting of 0.3 M Tris-HCl (pH 6.8), 25% 2-mercapto-ethanol, 12% sodium dodecyl sulfate (SDS), 25 mM EDTA, 20% glycerol, and 0.1% bromophenol blue. The mixtures were boiled at 100 °C for 10 min, loaded onto a stacking gel, and then separated onto 12% SDS-polyacrylamide mini-gels at a constant current of 20 mA with gel electrophoresis (DYCZ-24EN, Liuyi Biotechnology Co., Ltd., Beijing, China) [15].

For Western blot analysis, proteins on the gel were electro-transferred onto a 45 μm immobile membrane (PVDF; Millipore Corp., Bedford, MA, USA) using a transfer buffer composed of 25 mM Tris-HCl (pH 8.9), 192 mM glycine, and 20% methanol with protein electro transfer (DYCZ-40A, Liuyi Biotechnology Co., Ltd., Beijing, China). The membranes were blocked with blocking solution (20 mM Tris-HCl pH 7.4, 125 mM NaCl, 0.2% Tween 20, 1% bovine serum albumin, and 0.1% sodium azide) at room temperature for 1 h.

The Heme Oxygenase 1 (Rabbit, bs-2075R) was purchased from Bioss company (Bioss Biotechnology Co., Ltd., Beijing, China), and diluted 500-fold to be used. β-actin was purchased from proteintech (Proteintech Group, Inc., Rosemont, IL, USA), and diluted 4000-fold to be used. Following blocking, the membranes were incubated overnight with primary antibodies against heme oxygenase-1 (HO-1) at 4 °C. After washing three times (10 min each time) with 0.2% TPBS (0.2% Tween 20/PBS), the membranes were probed with appropriate secondary antibodies for 1 h at room temperature. Protein bands were visualized using enhanced chemiluminescence (ECL, Amersham Biosciences, Little Chalfont, UK). Band densities were quantified using a computer densitometer (AlphaImager 2200 System). Finally, all membranes were stripped and re-probed for β-Actin, serving as a loading control.

### 2.7. Statistical Analysis

All experiments were conducted in triplicate, and the data are presented as mean values ± standard deviation. One-way analysis of variance (ANOVA) followed by post-hoc tests to determine statistical significance between groups was performed. Tukey’s honestly significant difference (HSD) test was utilized as the post-hoc test to compare multiple group means. All experiments were conducted in triplicate, and the data were presented as mean values ± standard deviation. Statistical analysis was performed using a statistical software program (SPSS 22.0, Chicago, IL, USA). A *p*-value of less than 0.05 was considered to indicate a statistically significant difference.

## 3. Results and Discussion

### 3.1. Fractionation of RPTE Using Gel Filtration

**Figure 1:**
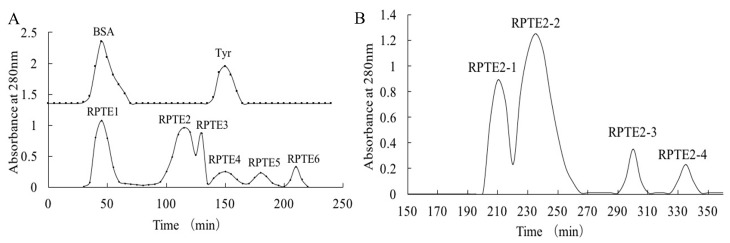
Gel filtration chromatography of RPTE. (**A**) Separation of RPTE fractions (RPTE1–RPTE6) by Sephadex G-25 column chromatography. (**B**) Sephadex G-15 column chromatography of RPTE2 fraction, resulting in the separation of RPTE2-1 to RPTE2-4 sub-fractions.

Gel filtration chromatography, a widely employed technique for separating bioactive substances based on molecular size, has been instrumental in purifying components from protein hydrolysates and removing impurities such as salts or buffer exchange [16,36,37,38]. In our study, we initially purified the hydrolysate RPTE using Sephadex G-25 chromatography. Concurrently, bovine serum albumin (66.4 kDa) and tyrosine (181.2 Da) were eluted from the column to serve as reference points. As Figure 1A depicts, six distinct fractions (RPTE1-RPTE6) were obtained from the RPTE hydrolysate. Based on molecular weight considerations, RPTE1 likely contained molecules larger than 50 kDa, while RPTE5 and RPTE6 were likely composed of small amino acids (<200 Da). It is noteworthy that peptides within the molecular size range of 0.5–3 kDa are often attributed to significant antioxidant activity in protein hydrolysates [19,39,40].

Subsequent assessment of free radical scavenging activity revealed that fractions with appropriate molecular weights (RPTE2, RPTE3, and RPTE4) exhibited robust scavenging capabilities (Table 1). Notably, RPTE2 displayed the most potent free radical scavenging activity, with IC_50_ values of 1.139 mg/mL for DPPH, 1.297 mg/mL for HO•, and 1.573 mg/mL for O_2_•^−^. Therefore, RPTE2, exhibiting the strongest antioxidant activity, was subjected to further purification using Sephadex G-15 column chromatography. As Figure 1B illustrates, RPTE2 was fractionated into four sub-fractions (RPTE2-1–RPTE2-4). Among these, RPTE2-2 demonstrated the most pronounced free radical scavenging activities. The IC_50_ values for RPTE2-2 in scavenging DPPH, HO•, and O_2_•^−^ were 1.486 mg/mL, 0.554 mg/mL, and 1.153 mg/mL, respectively (Table 2).

### 3.2. Isolation and Identification of Peptides from RPTE2-2 Fraction

**Figure 2:**
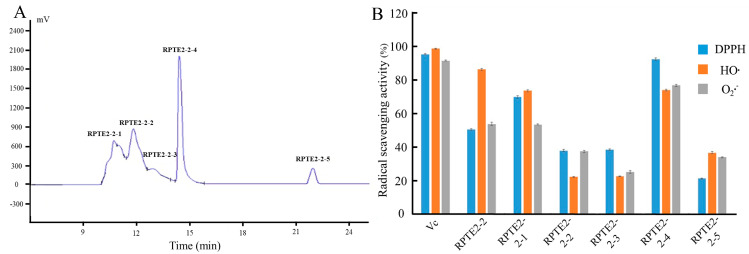
RP-HPLC separation of RPTE2-2 fraction and evaluation of its antioxidant activities. (**A**) RP-HPLC chromatogram showing the separation of RPTE2-2 into fractions RPTE2-2-1 to RPTE2-2-5. (**B**) Comparison of DPPH•, HO•, and O_2_•^−^ scavenging activities of RPTE2-2-4 fraction with Vitamin C.

As Figure 2A depicts, RPTE2-2, exhibiting robust free radical scavenging activities, was further fractionated into five fractions (RPTE2-2-1–RPTE2-2-5) via RP-HPLC using a linear gradient of acetonitrile. To assess the antioxidant activities of the fractions derived from RPTE2-2, scavenging activities against DPPH•, HO•, and O_2_•^–^ were determined at a concentration of 1.6 mg/mL and compared with Vitamin C at the same concentration. Notably, RPTE2-2-4 demonstrated DPPH•, HO•, and O_2_•^−^ scavenging ratios of 92.42%, 74.1%, and 74.8%, respectively, as Figure 2B illustrates. RPTE2-2-4 exhibited antioxidant activity comparable to that of Vitamin C.

**Figure 3:**
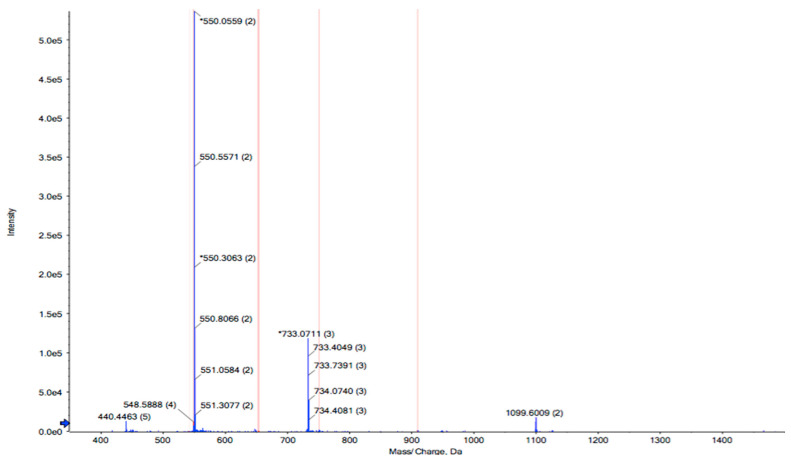
Identification of the peptide sequence from RPTE2-2-4 by LC-MS/MS analysis. The peptide sequence Leu-Phe-Lys-Lys-Asn-Leu-Leu-Thr-Leu (LFKKNLLTL) was determined.

RPTE2-2-4, characterized by stronger free radical scavenging activities, was identified using LC-MS/MS combined with the MASCOT V2.3 search engine. The amino acid sequence of RPTE2-2-4 was determined to be Leu-Phe-Lys-Lys-Asn-Leu-Leu-Thr-Leu (LFKKNLLTL) with a molecular weight of 1089.39 Da (Figure 3). Previous studies have indicated that the antioxidant properties of peptides are influenced by various factors, including molecular weight, amino acid sequence, and hydrophobicity of the constituent amino acids [41,42,43,44]. Antioxidative peptides typically exhibit structural characteristics such as relatively short peptide lengths (e.g., 3–16 amino acids), molecular weights ranging from 500 to 1800 Da, and the presence of hydrophobic amino acid residues in the sequence [45]. The presence of aromatic amino acids and/or hydrophobic amino acids, such as Tyr, His, Trp, Phe, Val, Leu, and Ala, has been suggested to be crucial for the antioxidant activities of peptides [45,46,47].

The isolated peptide (LFKKNLLTL) is relatively short and contains an aromatic amino acid (Phe) and four hydrophobic amino acid (Leu) residues in the sequence, aligning closely with these structural characteristics. In addition to having hydrophobic amino acids such as Leu or Val in their N-terminal regions, protein hydrolyzates, and peptides containing the nucleophilic sulfur-containing amino acid residues (Cys and Met), aromatic amino acid residues (Phe, Trp, and Tyr), and/or the imidazole ring-containing His have been generally found to possess strong antioxidant properties [47]. The presence and positioning of specific amino acids within the peptide structure play a crucial role in determining its antioxidant properties. Specifically, the aromatic amino acid phenylalanine (Phe) and the hydrophobic amino acid leucine (Leu) residues are strategically positioned within the peptide sequence. Phenylalanine is known for its ability to donate electrons and stabilize free radicals through resonance, thereby neutralizing their harmful effects. Additionally, leucine residues may contribute to the hydrophobicity of the peptide, allowing it to interact with lipid membranes and scavenge lipid peroxides. This alignment between the peptide’s structure and the speculated requirements for antioxidant activity is consistent with the results of our in vitro free radical scavenging assays. Therefore, the synthesized peptide was further investigated for its antioxidant activities in a HepG2 cell system.

### 3.3. Cellular Antioxidant Effects of Peptide in HepG2 Cells under Oxidative Stress

#### 3.3.1. Cell Viability

**Figure 4:**
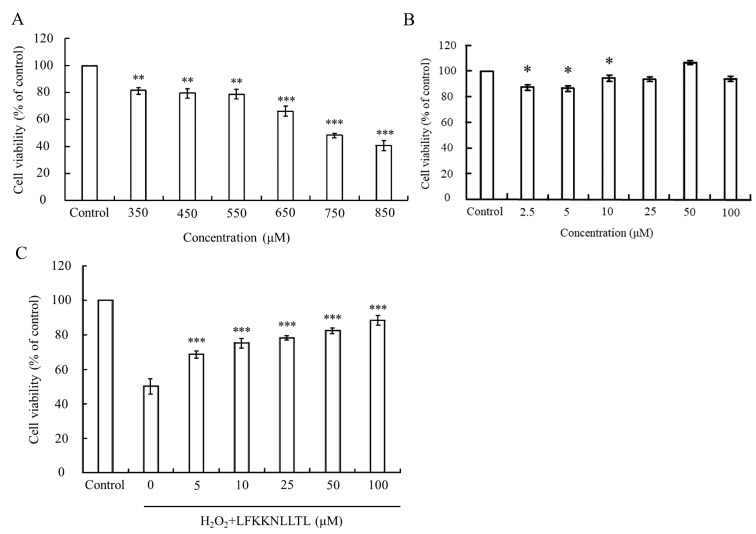
Evaluation of cell viability in HepG2 cells under oxidative stress and peptide treatment. (**A**) Viability of HepG2 cells treated with varying concentrations of H_2_O_2_. (**B**) Viability of HepG2 cells treated with different concentrations of the peptide. (**C**) Protective effect of the peptide against H_2_O_2_-induced cytotoxicity in HepG2 cells. The group without peptide and H_2_O_2_ treatment served as the blank. Data were presented as mean ± SD. Statistical significance was denoted as * *p* < 0.05, ** *p* < 0.01, and *** *p* < 0.001 compared to the blank in (**A**,**B**), and *** *p* < 0.001 compared to the 750 μM H_2_O_2_ treatment group in (**C**).

In this study, the MTT assay was employed to assess oxidative stress-induced cytotoxicity, measuring the metabolic activity of cells via the oxidation-reduction activities of the mitochondria, which involve the mitochondrial succinate dehydrogenase system. The viability of HepG2 cells treated solely with various concentrations of H_2_O_2_ or pre-incubated with the peptide (LFKKNLLTL) with or without H_2_O_2_-induced oxidative stress is depicted in Figure 4.

H_2_O_2_ is known to directly inflict damage on DNA, lipids, and other macromolecules, leading to oxidative injury to cells [48,49]. As Figure 4A illustrates, the viability of HepG2 cells incubated with different concentrations of H_2_O_2_ declined in a dose-dependent manner. A significant difference (*p* < 0.05) compared to the control group was observed when the concentration of H_2_O_2_ exceeded 350 μM. Upon reaching a concentration of 750 μM, the viability of HepG2 cells plummeted to approximately 50%, indicating severe damage caused by excessive H_2_O_2_. However, treatment with the peptide did not induce any cytotoxic effects on HepG2 cells at the tested concentrations (Figure 4B). Notably, pretreatment with the peptide significantly (*p* < 0.001) reduced the cytotoxicity induced by 750 μM H_2_O_2_. As Figure 4C depicts, the peptide exhibited a protective effect against H_2_O_2_-induced cell damage in a dose-dependent manner. Cell viability reached up to 88.3% when the pretreatment concentration of the peptide increased to 100 μM. These findings suggest that the peptide has the potential to mitigate the cytotoxic effects induced by oxidative stress, thereby preserving cell viability.

#### 3.3.2. Effect of the Peptide on Intracellular ROS and GSH Levels

The production of reactive oxygen species (ROS) and the depletion of glutathione (GSH) are interconnected events that influence various signal transduction pathways regulating cell survival. Elevated ROS levels, coupled with decreased intracellular GSH content, contribute to mitochondrial dysfunction [50,51]. To elucidate the defense mechanism of the peptide against H_2_O_2_-induced cell injury in HepG2 cells, we assessed intracellular ROS and GSH levels using DCFH-DA and CMFDA, respectively.

**Figure 5:**
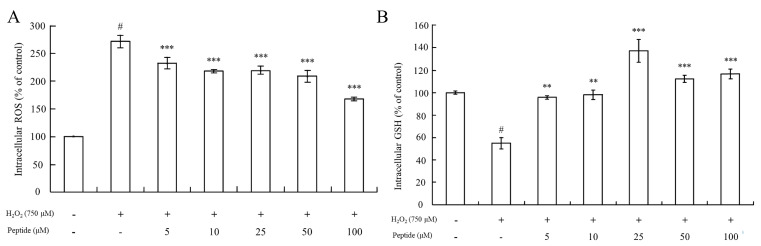
Measurement of intracellular ROS levels and GSH content in HepG2 cells upon peptide treatment under oxidative stress. (**A**) Changes in intracellular ROS levels in HepG2 cells under oxidative stress and peptide treatment. (**B**) GSH content in HepG2 cells treated with the peptide under oxidative stress conditions. Data were presented as mean ± SD. Statistical significance was indicated as # *p* < 0.001 compared to the blank, ** *p* < 0.01, and *** *p* < 0.001 compared to the H_2_O_2_ treatment group.

As Figure 5A depicts, intracellular ROS levels in HepG2 cells treated with 750 μM H_2_O_2_ increased significantly (*p* < 0.001) compared to controls. However, there was a significant difference in intracellular ROS levels between the HepG2 cell groups pretreated with or without the peptide before exposure to oxidative stress induced by 750 μM H_2_O_2_. Pretreatment with different concentrations of the peptide for 2 h resulted in significantly decreased intracellular ROS levels compared to HepG2 cells treated only with H_2_O_2_ (*p* < 0.001). This suggests that the peptide possesses the capacity to eliminate ROS within the cells.

Furthermore, the determination of GSH levels showed that pretreatment of cells with the peptide before oxidative stimuli led to an increase in GSH levels compared to cells treated with 750 μM H_2_O_2_ alone (stress control) (Figure 5B). In fact, GSH levels even surpassed those of the basal line of the blank group (without H_2_O_2_ treatment) when cells were pretreated with the peptide at concentrations of 25 μM or higher. This suggests that pretreatment with a sufficient concentration of this peptide could enhance the GSH/GSSG ratios in the cells, thereby increasing GSH levels. Guofu Yi determined the effects of soybean protein hydrolysates against intracellular antioxidant activity, and also found that soybean peptides inhibited the production of oxidized glutathione (GSSG) in HepG2 cells [52]. Furthermore, Ning Li explored the protecting-efficacies of wheat germ peptides to PC12 cells from lead-induced oxidative stress [53]. In summary, our findings indicate that the peptide exhibits antioxidant properties by reducing intracellular ROS levels and enhancing GSH levels, thereby contributing to cellular defense mechanisms against oxidative stress-induced injury in HepG2 cells.

#### 3.3.3. Influence of the Peptide on HO-1 Activation

**Figure 6:**
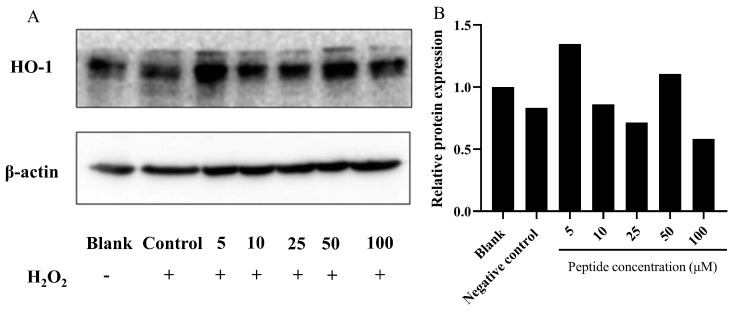
Analysis of HO-1 expression in HepG2 cells treated with the peptide under oxidative stress. (**A**) Representative images; (**B**) Quantitative results. Expression of HO-1 protein in HepG2 cells pretreated with the peptide and exposed to H_2_O_2_-induced oxidative stress. The group without peptide and H_2_O_2_ treatment served as the blank, while the 750 μM H_2_O_2_ treatment group was designated as the control. The numbers at the bottom of the figure indicate the peptide at concentrations of 5, 10, 25, 50, and 100 μM.

Heme oxygenase-1 (HO-1) is recognized as a cytoprotective protein, the expression of which is consistently linked to therapeutic benefits in various pathological conditions [54]. To elucidate the cytoprotective mechanism against oxidative stress induced by H_2_O_2_ in HepG2 cells, we analyzed the expression of phase II detoxifying enzymes such as HO-1 via Western blot analysis. As Figure 6 depicts, compared to the blank group (without H_2_O_2_ and peptide), HO-1 expression was slightly induced by treatment with H_2_O_2_ alone. However, pretreatment with the peptide in HepG2 cells notably upregulated the expression of HO-1. This suggests that the peptide could mitigate oxidative stress induced by H_2_O_2_ by stimulating HO-1 expression. Notably, similar actions of peptides isolated from *M. edulis* protein or sardine muscle on HO-1 expression have been extensively discussed in previous studies [55,56].

## 4. Conclusions

In summary, our study comprehensively investigated the antioxidant properties of a peptide isolated from the hydrolysate of Manila clam (*Ruditapes philippinarum*) muscle. Through a series of purification steps, we successfully identified a peptide fraction, designated as RPTE2-2-4, which exhibited potent free radical scavenging activities against DPPH•, HO•, and O_2_•^−^. This fraction demonstrated antioxidant efficacy comparable to Vitamin C, indicating its potential as a natural antioxidant agent. Further characterization of the isolated peptide, LFKKNLLTL, revealed its structural features, including a relatively short peptide length and the presence of aromatic and hydrophobic amino acid residues, which are known to contribute to antioxidant activity. Notably, our study also demonstrated the protective effects of this peptide against H_2_O_2_-induced oxidative stress in HepG2 cells. Pretreatment with the peptide resulted in a dose-dependent reduction in intracellular ROS levels and elevation of GSH levels, highlighting its ability to modulate cellular defense mechanisms against oxidative damage.

Moreover, our findings indicated that the peptide could stimulate the expression of the cytoprotective enzyme HO-1, further reinforcing its antioxidant properties and potential therapeutic benefits. This observation aligns with previous studies highlighting the role of HO-1 induction in cellular protection against oxidative stress. Overall, our study underscores the potential of the Manila clam-derived peptide as a promising natural antioxidant agent with cytoprotective properties. Further exploration of its mechanisms of action and in vivo efficacy could provide valuable insights for the development of novel therapeutic interventions targeting oxidative stress-related diseases. While bioactive peptides hold promise as potential therapeutic agents, addressing these limitations is crucial for their successful translation into clinically viable treatments for oxidative stress-related diseases. Further research and development efforts are needed to optimize their formulation, enhance bioavailability, ensure safety, and navigate regulatory hurdles.

## Figures and Tables

**Table 1:** IC_50_ (mg/mL) of RPTE fractions (RPTE1 to RPTE6) from Sephadex G-25 separation on DPPH•, HO•, and O_2_•^−^ scavenging activities.

Fractions	DPPH•	HO•	O_2_•^−^
RPTE1	50.951 ± 5.931 ^a^	5.773 ± 0.057 ^b^	193.803 ± 59.407 ^a^
RPTE2	1.139 ± 0.002 ^d^	1.297 ± 0.023 ^b^	1.573 ± 0.006 ^c^
RPTE3	1.695 ± 0.005 ^d^	2.693 ± 0.023 ^b^	5.147 ± 0.238 ^bc^
RPTE4	2.700 ± 0.017 ^cd^	2.633 ± 0.038 ^b^	4.743 ± 0.162 ^bc^
RPTE5	35.473 ± 0.311 ^b^	86.817 ± 19.312 ^a^	31.860 ± 0.320 ^b^
RPTE6	6.950 ± 0.036 ^c^	6.980 ± 0.506 ^b^	15.220 ± 3.852 ^bc^

Note: IC_50_ was defined as the concentration where a sample caused a 50% decrease of the initial concentration of DPPH•, HO• and O_2_•^−^, respectively. All data are presented as the mean ± SD of triplicate results. Different lowercase letters indicate a significant difference at the 0.05 level.

**Table 2:** IC_50_ (mg/mL) of fractions (RPTE2-1 to RPTE 2-4) from Sephadex G-15 separation on DPPH•, HO•, and O_2_•^−^ scavenging activities.

Fractions	DPPH•	HO•	O_2_•^−^
RPTE2-1	2.581 ± 0.179 ^b^	1.043 ± 0.012 ^c^	14.293 ± 2.005 ^b^
RPTE2-2	1.486 ± 0.014 ^c^	0.554 ± 0.010 ^d^	1.153 ± 0.023 ^d^
RPTE2-3	5.211 ± 1.042 ^b^	2.247 ± 0.015 ^b^	7.337 ± 1.689 ^c^
RPTE2-4	12.710 ± 6.066 ^a^	6.373 ± 0.278 ^a^	18.677 ± 5.859 ^a^

Note: IC_50_ was defined as the concentration where a sample caused a 50% decrease of the initial concentration of DPPH•, HO• and O_2_•^−^, respectively. All data are presented as the mean ± SD of triplicate results. Different lowercase letters indicate a significant difference at the 0.05 level.

## Data Availability

The original contributions presented in the study are included in the article, further inquiries can be directed to the corresponding authors.

## References

[1] Mansoor S., Ali Wani O., Lone J.K., Manhas S., Kour N., Alam P., Ahmad A., Ahmad P. (2022). Reactive oxygen species in plants: From source to sink. Antioxidants.

[2] Bhattacharjee S., Bhattacharjee S. (2019). ROS and oxidative stress: Origin and implication. Reactive Oxygen Species in Plant Biology.

[3] Adwas A.A., Elsayed A., Azab A., Quwaydir F. (2019). Oxidative stress and antioxidant mechanisms in human body. J. Appl. Biotechnol. Bioeng.

[4] Tan B.L., Norhaizan M.E., Liew W.-P.-P., Sulaiman Rahman H. (2018). Antioxidant and oxidative stress: A mutual interplay in age-related diseases. Front. Pharmacol..

[5] Pacinella G., Ciaccio A.M., Tuttolomondo A. (2022). Endothelial Dysfunction and Chronic Inflammation: The Cornerstones of Vascular Alterations in Age-Related Diseases. Int. J. Mol. Sci..

[6] Pyo I.S., Yun S., Yoon Y.E., Choi J.-W., Lee S.-J. (2020). Mechanisms of aging and the preventive effects of resveratrol on age-related diseases. Molecules.

[7] Martemucci G., Portincasa P., Di Ciaula A., Mariano M., Centonze V., D’Alessandro A.G. (2022). Oxidative stress, aging, antioxidant supplementation and their impact on human health: An overview. Mech. Ageing Dev..

[8] Akbari B., Baghaei-Yazdi N., Bahmaie M., Mahdavi Abhari F. (2022). The role of plant-derived natural antioxidants in reduction of oxidative stress. BioFactors.

[9] Gulcin İ. (2020). Antioxidants and antioxidant methods: An updated overview. Arch. Toxicol..

[10] Anwar H., Hussain G., Mustafa I. (2018). Antioxidants from natural sources. Antioxid. Foods Its Appl..

[11] Hamidi M., Kozani P.S., Kozani P.S., Pierre G., Michaud P., Delattre C. (2019). Marine bacteria versus microalgae: Who is the best for biotechnological production of bioactive compounds with antioxidant properties and other biological applications?. Mar. Drugs.

[12] Okoye C.O., Ezeorba T.P., Okeke E.S., Okagu I.U. (2022). Recent findings on the isolation, identification and quantification of bioactive peptides. Appl. Food Res..

[13] Görgüç A., Gençdağ E., Yılmaz F.M. (2020). Bioactive peptides derived from plant origin by-products: Biological activities and techno-functional utilizations in food developments—A review. Food Res. Int..

[14] Cunha S.A., Pintado M.E. (2022). Bioactive peptides derived from marine sources: Biological and functional properties. Trends Food Sci. Technol..

[15] Cheng S., Tu M., Chen H., Xu Z., Wang Z., Liu H., Zhao G., Zhu B., Du M. (2018). Identification and inhibitory activity against α-thrombin of a novel anticoagulant peptide derived from oyster (*Crassostrea gigas*) protein. Food Funct..

[16] Cheng S., Tu M., Liu H., An Y., Du M., Zhu B. (2021). A novel heptapeptide derived from *Crassostrea gigas* shows anticoagulant activity by targeting for thrombin active domain. Food Chem..

[17] Manzoor M., Singh J., Gani A. (2022). Exploration of bioactive peptides from various origin as promising nutraceutical treasures: In vitro, in silico and in vivo studies. Food Chem..

[18] Lorenzo J.M., Munekata P.E., Gómez B., Barba F.J., Mora L., Pérez-Santaescolástica C., Toldrá F. (2018). Bioactive peptides as natural antioxidants in food products—A review. Trends Food Sci. Technol..

[19] Sila A., Bougatef A. (2016). Antioxidant peptides from marine by-products: Isolation, identification and application in food systems. A review. J. Funct. Foods.

[20] Zhang X., Cao D., Sun X., Sun S., Xu N. (2019). Preparation and identification of antioxidant peptides from protein hydrolysate of marine alga Gracilariopsis lemaneiformis. J. Appl. Phycol..

[21] Sampath Kumar N., Nazeer R., Jaiganesh R. (2012). Purification and identification of antioxidant peptides from the skin protein hydrolysate of two marine fishes, horse mackerel (*Magalaspis cordyla*) and croaker (*Otolithes ruber*). Amino Acids.

[22] Gökoğlu N., Gökoğlu N. (2021). Molluscan Shellfish. Shellfish Processing and Preservation.

[23] Heffernan S., Giblin L., O’Brien N. (2021). Assessment of the biological activity of fish muscle protein hydrolysates using in vitro model systems. Food Chem..

[24] Macedo M.W.F.S., Cunha N.B.D., Carneiro J.A., Costa R.A.D., Alencar S.A.D., Cardoso M.H., Franco O.L., Dias S.C. (2021). Marine organisms as a rich source of biologically active peptides. Front. Mar. Sci..

[25] Ahmmed M.K., Bhowmik S., Giteru S.G., Zilani M.N.H., Adadi P., Islam S.S., Kanwugu O.N., Haq M., Ahmmed F., Ng C.C.W. (2022). An update of lectins from marine organisms: Characterization, extraction methodology, and potential biofunctional applications. Mar. Drugs.

[26] Kiiskinen L.-L., Viikari L., Kruus K. (2002). Purification and characterisation of a novel laccase from the ascomycete Melanocarpus albomyces. Appl. Microbiol. Biotechnol..

[27] Yiasmin M.N., Islam M.S., He H., Liu Y., Wang M., Yang R., Hua X. (2020). Purification, isolation, and structure characterization of water soluble and insoluble polysaccharides from Maitake fruiting body. Int. J. Biol. Macromol..

[28] Sánchez J.C., García R.F., Cors M.T.M. (2010). 1, 1-Diphenyl-2-picrylhydrazyl radical and superoxide anion scavenging activity of *Rhizophora mangle* (L.) bark. Pharmacogn. Res..

[29] Xia Y., Ma C., Ju R., Zhao C., Zheng F., Sun X., Li Z., Wang C., Shi D., Lin X. (2021). Characterization of nitrogen-containing compounds in petroleum fractions by online reversed-phase liquid chromatography-electrospray ionization Orbitrap mass spectrometry. Fuel.

[30] Yang J., Huang J., Dong X., Zhang Y., Zhou X., Huang M., Zhou G. (2020). Purification and identification of antioxidant peptides from duck plasma proteins. Food Chem..

[31] Pan X., Zhao Y.Q., Hu F.Y., Wang B. (2016). Preparation and identification of antioxidant peptides from protein hydrolysate of skate (*Raja porosa*) cartilage. J. Funct. Foods.

[32] Wang L., Gao Y., Li J., Subirade M., Song Y., Liang L. (2016). Effect of resveratrol or ascorbic acid on the stability of alpha-tocopherol in O/W emulsions stabilized by whey protein isolate: Simultaneous encapsulation of the vitamin and the protective antioxidant. Food Chem..

[33] Yang P., Ke H., Hong P., Zeng S., Cao W. (2011). Antioxidant activity of bigeye tuna (*Thunnus obesus*) head protein hydrolysate prepared with Alcalase. Int. J. Food Sci. Technol..

[34] Cho B.-O., Ryu H.-W., Lee C.-W., Jin C.-H., Seo W.-D., Ryu J., Kim D.-S., Kang S.Y., Yook H.-S., Jeong I.-Y. (2015). Protective effects of new blackberry cultivar MNU-32 extracts against H2O2-induced oxidative stress in HepG2 cells. Food Sci. Biotechnol..

[35] Yeh C.C., Yang J.I., Lee J.C., Tseng C.N., Chan Y.C., Hseu Y.C., Tang J.Y., Chuang L.Y., Huang H.W., Chang F.R. (2012). Anti-proliferative effect of methanolic extract of *Gracilaria tenuistipitata* on oral cancer cells involves apoptosis, DNA damage, and oxidative stress. BMC Complem. Altern. M.

[36] Mahato N., Sinha M., Sharma K., Koteswararao R., Cho M.H. (2019). Modern extraction and purification techniques for obtaining high purity food-grade bioactive compounds and value-added co-products from citrus wastes. Foods.

[37] Jayaprakash R., Perera C.O. (2020). Partial purification and characterization of bioactive peptides from cooked New Zealand green-lipped mussel (*Perna canaliculus*) protein hydrolyzates. Foods.

[38] Cheng S., Wu D., Yuan L., Liu H., Ei-Seedi H.R., Du M. (2022). *Crassostrea gigas*-Based Bioactive Peptide Protected Thrombin-Treated Endothelial Cells against Thrombosis and Cell Barrier Dysfunction. J. Agric. Food Chem..

[39] Nasri M. (2017). Protein hydrolysates and biopeptides: Production, biological activities, and applications in foods and health benefits. A review. Adv. Food Nutr. Res..

[40] Olivares-Galván S., Marina M.L., García M.C. (2020). Extraction and characterization of antioxidant peptides from fruit residues. Foods.

[41] Ghribi A.M., Sila A., Przybylski R., Nedjar-Arroume N., Makhlouf I., Blecker C., Attia H., Dhulster P., Bougatef A., Besbes S. (2015). Purification and identification of novel antioxidant peptides from enzymatic hydrolysate of chickpea (*Cicer arietinum* L.) protein concentrate. J. Funct. Foods.

[42] Escudero E., Mora L., Fraser P.D., Aristoy M.C., Toldra F. (2013). Identification of novel antioxidant peptides generated in Spanish dry-cured ham. Food Chem..

[43] Li Y.-W., Li B. (2013). Characterization of structure–antioxidant activity relationship of peptides in free radical systems using QSAR models: Key sequence positions and their amino acid properties. J. Theor. Biol..

[44] Mirzaee H., Ahmadi Gavlighi H., Nikoo M., Udenigwe C.C., Khodaiyan F. (2023). Relation of amino acid composition, hydrophobicity, and molecular weight with antidiabetic, antihypertensive, and antioxidant properties of mixtures of corn gluten and soy protein hydrolysates. Food Sci. Nutr..

[45] Samaranayaka A.G.P., Li-Chan E.C.Y. (2011). Food-derived peptidic antioxidants: A review of their production, assessment, and potential applications. J. Funct. Foods.

[46] Ngo D.-H., Qian Z.-J., Ryu B., Park J.W., Kim S.-K. (2010). In vitro antioxidant activity of a peptide isolated from Nile tilapia (*Oreochromis niloticus*) scale gelatin in free radical-mediated oxidative systems. J. Funct. Foods.

[47] Nwachukwu I.D., Aluko R.E. (2019). Structural and functional properties of food protein-derived antioxidant peptides. J. Food Biochem..

[48] Juan C.A., Pérez de la Lastra J.M., Plou F.J., Pérez-Lebeña E. (2021). The chemistry of reactive oxygen species (ROS) revisited: Outlining their role in biological macromolecules (DNA, lipids and proteins) and induced pathologies. Int. J. Mol. Sci..

[49] Sharma A., Gupta P., Prabhakar P.K. (2019). Endogenous repair system of oxidative damage of DNA. Curr. Chem. Biol..

[50] Liu T., Sun L., Zhang Y., Wang Y., Zheng J. (2022). Imbalanced GSH/ROS and sequential cell death. J. Biochem. Mol. Toxicol..

[51] Marí M., de Gregorio E., de Dios C., Roca-Agujetas V., Cucarull B., Tutusaus A., Morales A., Colell A. (2020). Mitochondrial glutathione: Recent insights and role in disease. Antioxidants.

[52] Yi G., ud Din J., Zhao F., Liu X. (2020). Effect of soybean peptides against hydrogen peroxide induced oxidative stress in HepG2 cells via Nrf2 signaling. Food Funct..

[53] Li N., Wen L., Wang F., Wang T., Li T., Qiao M., Song L., Bukyei E., Huang X. (2022). Mechanism of mitigating effect of wheat germ peptides on lead-induced oxidative damage in PC12 cells. Ecotoxicol. Environ. Saf..

[54] Consoli V., Sorrenti V., Grosso S., Vanella L. (2021). Heme oxygenase-1 signaling and redox homeostasis in physiopathological conditions. Biomolecules.

[55] Park S.Y., Kim Y.S., Ahn C.B., Je J.Y. (2016). Partial purification and identification of three antioxidant peptides with hepatoprotective effects from blue mussel (*Mytilus edulis*) hydrolysate by peptic hydrolysis. J. Funct. Foods.

[56] Erdmann K., Grosser N., Schipporeit K., Schroder H. (2006). The ACE inhibitory dipeptide met-tyr diminishes free radical formation in human endothelial cells via induction of heme oxygenase-1 and ferritin. J. Nutr..

